# Development of a Multiwavelength Visible-Range-Supported Opto–Ultrasound Instrument Using a Light-Emitting Diode and Ultrasound Transducer

**DOI:** 10.3390/s18103324

**Published:** 2018-10-03

**Authors:** Hojong Choi, Jung-Yeol Yeom, Jae-Myung Ryu

**Affiliations:** 1 Department of Medical IT Convergence Engineering, Kumoh National Institute of Technology, Gumi 39253, Korea; hojongch@kumoh.ac.kr; 2 School of Biomedical Engineering, Korea University, Seoul 02841, Korea; 3 Department of Optical Engineering, Kumoh National Institute of Technology, Gumi 39253, Korea

**Keywords:** visible light, multiwavelength, light-emitting diode, ultrasound transducer

## Abstract

A new multiwavelength visible-range-supported opto–ultrasound instrument using a light-emitting diode and ultrasound transducer was developed in order to produce multiwavelength visible light with minimized color aberration errors, and detect ultrasound signals emitted from the target. In the instrument, the developed optical systems can provide multiwavelength optical transmission with low optical aberration within 10-cm ranges that are reasonably flat in the modulation transfer function at spatial frequencies of 20 and 40 lp/mm, except at the end of the diagonal edge of the samples. To assess the instrument capability, we performed pulse–echo responses with *Thunnus obesus* eye samples. Focused red, green, blue and white light rays from an integrated red, green and blue LED source were produced, and echo signal amplitudes of 33.53, 34.92, 38.74 and 82.54 mV, respectively, were detected from the *Thunnus obesus* eye samples by a 10-MHz focused ultrasound transducer. The center frequencies of the echo signal when producing red, green, blue and white LED light in the instrument were 9.02, 9.05, 9.21 and 8.81 MHz, respectively. From these tests, we verify that this instrument can combine red, green and blue LED light to cover different wavelengths in the visible-light range and detect reasonable echo amplitudes from the samples.

## 1. Introduction

Ultrasound systems have been used to obtain structural information from samples in nondestructive and medical applications, and optical systems have been utilized to obtain functional information in animal and biological applications [1,2,3]. Ultrasound systems have limited penetration depth as the operating frequency of the ultrasound increases; however, higher-frequency ultrasound systems provide relatively higher spatial resolution compared to lower-frequency ultrasound systems by sacrificing penetration depth [4,5,6]. On the other hand, optical systems typically provide high contrast information and relatively low spatial resolution of samples due to light scattering while generating comparatively low power compared to only ultrasound systems [7,8].

Thus, hybrid systems that combine optical systems as transmitters and ultrasound systems as receivers are being highlighted to utilize advantages of both modalities, such as good contrast and spatial resolution attributed to light and ultrasound signal properties [9,10]. Opto–ultrasound instruments are being widely used for a variety of the biological applications that follow: one potential application is human-skull imaging using a photon recycler to improve penetration depth of the light [11], while using an opto–ultrasound instrument, myocardial infraction, brain and the whole body of the mouse were also obtained for preclinical research [12,13,14]. Opto–ultrasound instruments could also provide tissue information about hemoglobin, lipids and blood oxygenation [7].

An opto–ultrasound instrument, however, still provides limited functional information at a single wavelength of light. Different tissues or cells such as hemoglobin, collagen and DNA are known to show different optical absorptions at different wavelengths [10]. The peak absorptions of oxyhemoglobin and deoxyhemoglobin are at ~400 and ~600 nm, respectively, whereas those of water are at ~800 and ~1000 nm [15,16]. Compared to hemoglobin and water, melanin has no absorption peak and is absorbed in the lower wavelength ranges between 300 and 800 nm [15]. Each tissue has its own absorption spectra at different wavelength ranges [15]. Therefore, the 400 and 600 nm optical wavelength range may be useful for hemoglobin and melanin tissue without water.

In optical imaging or therapy applications, lasers are the standard transmission sources [17,18]. Compared to lasers, LEDs’ divergent light property can critically lower detection sensitivity [19]. In addition, a typical LED-based system requires the use of a more complex optical lens setup compared to a laser-based system [20]. The intensity of the LED light is also much lower than the laser [20]. Therefore, the received echo signals when using an LED are relatively lower than when using a laser such that ultrasound transducers with low sensitivity are not suitable for opto–ultrasound instruments. To increase the intensity of the LED lights, the LED array structures could be constructed [20]. Using array-type LED sources, however, also requires optical lenses for focusing the light beam on the desired spots. Another limitation of an LED-based system is that the LED itself is typically limited in wavelength bandwidth such that several LED light sources need to be mixed when producing multiwavelength LED systems [20]. In addition, LED lights are not purely monochromatic and possess a small range of wavelengths compared to lasers [19]. Therefore, an opto–acoustic multiwavelength system using a laser has been developed [21]. However, compared to lasers, LEDs are a relatively harmless, cost-effective, portable and compact excitation source [20]. An opto–acoustic instrument using an LED with low optical aberration has not been designed. Therefore, in this study we develop a new type of LED–ultrasound (LED–US) instrument capable of a broad visible-light wavelength range with low optical aberration, because it is crucial to avoid optical aberration when combining different wavelengths of LED light.

In this paper, we present a new multiwavelength visible-range-supported opto–ultrasound instrument that is able to produce multiwavelength visible light with low color aberration for potential applications in ophthalmology. To utilize the adjustable focal distances in the optical system, mounts for attaching optical lenses can be placed in front of the transmission output points in the system. The optical system used in this study is an optical product called a “micro lens”. This product can be used for observing microscopic objects such as insects’ eyes and flowers [22]. The characteristics of such an optical system are high magnification and short object distances compared to other optical products. These features can be used to focus the LED light close to the samples. Therefore, this developed instrument can be easily applied to targets with different focal distances if desired.

## 2. Materials and Methods

**Figure 1:**
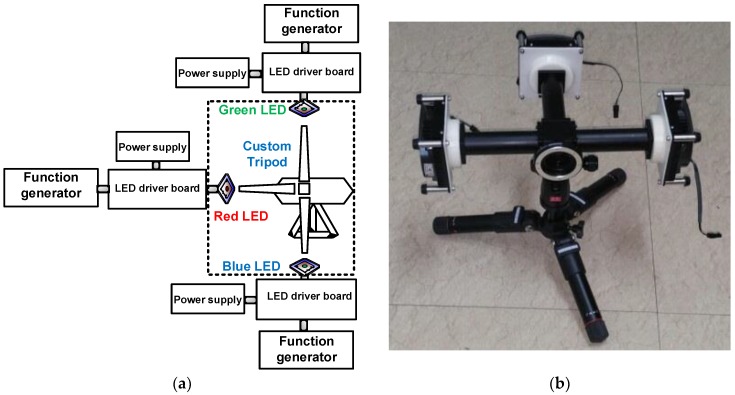
(**a**) Concept of the LED transmission system and (**b**) custom tripod with the LED and a heatsink.

To implement a multiwavelength visible-range-supported LED–US instrument using three visible colors such as red, green and blue LEDs, we fabricated a custom tripod. Figure 1 shows the concept of the LED system in the multiwavelength visible-range-supported LED–US instrument with the custom tripod. A heatsink (Aavid Thermalloy, San Jose, CA, USA) was attached to the bottom of each LED to effectively produce the light. The LED driver boards (DK-136M-3, Luminus Devices, Sunnyvale, CA, USA) were supported by an arbitrary function generator (AFG3252C, Tektronix Inc., Beaverton, OR, USA) and the switching DC power supply (PAS20-36, Kikusui Electronics Corp., Yokohama, Japan) provided DC power to the red, green and blue LEDs. A DC power supply was used to generate 1-kHz and 5-V_p-p_ pulses from an arbitrary function generator to trigger the LED driver boards to control the red, green and blue LEDs, respectively. The customized tripod was fabricated to integrate the light sources to generate light with low optical aberration for any desirable direction. Compared to commercial multiwavelength LED light products, the developed optical system can provide adjustable focus and power with more freedom. Having low optical aberration is an essential property in optical systems for generating high-resolution and broadband light. Therefore, guides between each LED and the optical components were added for guiding the LED light sources with the optical components. The guides to be connected between each LED source and the custom tripod were also fabricated using a commercial three-dimensional printer (Fortus450mc, Stratasys, Eden Prarie, MI, USA) to avoid possible light divergence inside the guides.

The fundamental design concept of the optical system used in this study is described in [22]. However, focusing is performed by moving two or more lens groups to the object distance at which the samples are placed. This process focuses the LED light on the samples. It is also necessary to calculate the illumination area at the samples. Therefore, we need to design a new optical system to focus the LED light on the samples.

**Figure 2:**
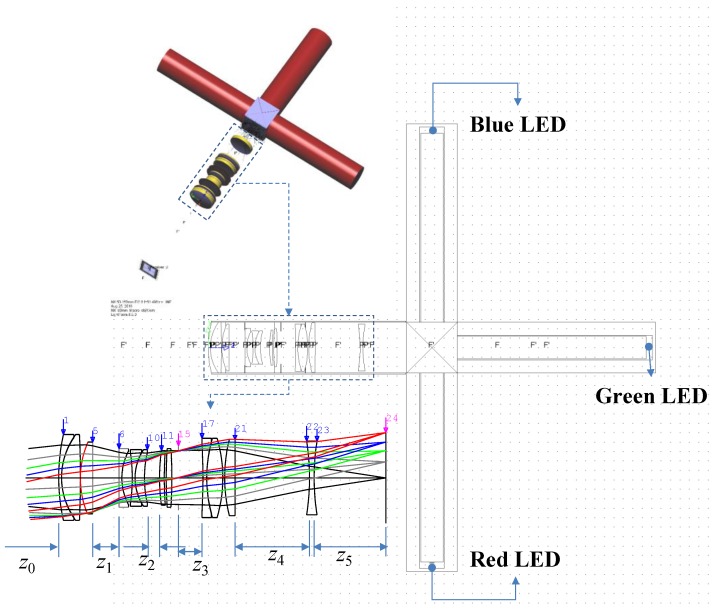
Optical layout for the multiwavelength visible-range-supported LED transmission system.

The optical path of the designed optical system used in this study is shown in Figure 2. The distance from the samples to the first surface of the optical system is 100 mm. The lens group that moves during focusing comprises the 6th to 11th planes and the 17th to 21st planes. The other lens groups are fixed at the focusing time. When focusing the light in the designed optical system, the two lens groups must move independently at the same time, so a CAM (a mechanical component that converts rotational motion into linear motion) should be used. One of the two lens groups undergoes a linear motion, and the other lens group undergoes a curved motion. Therefore, the distance from the object to the first surface (*z*_0_), the distance between the 5th and the 6th surfaces (*z*_1_), the distance between the 10th and the 11th surfaces (*z*_2_), the distance between the 15th and the 17th surfaces (*z*_4_), and the distance between the 21st and the 22nd surfaces all change during focusing. For focusing in the optical system, two lens groups move back and forth. For this purpose, CAM was applied to the focusing ring.

The distances with respect to the CAM angles are given in Table 1. Because the distance from the sample to the first surface of the optical system is 100 mm, the CAM angle will be between 60 and 70 degrees. Once the CAM angle is obtained, the illuminated area can be obtained by calculating the magnification.

Because the lens group from the 6th surface to the 10th surface is subjected to linear motion, the CAM angle and the distance between the 5th and 6th surfaces satisfies a linear equation. Because the distance from the 1st to the 11th surface is constant, the distance between the 10th and 11th surfaces can be easily determined. Similarly, because the distance from the 15th surface to the image is constant, if the distance between the 15th and 17th surfaces is known, the distance between the 21st and 22nd surfaces can be known.

If the effective focal length (EFL) of the lens group from the 1st to 5th surface is *f*_1_, the EFL of the lens group from the 6th to 10th surface is *f*_2_, the EFL of the lens group from the 11th to 14th surface is *f*_3_, the EFL of the lens group from the 17th to 21st surface is *f*_4_, and the EFL of the lens group from the 22nd to 23rd surface is *f*_5_, then the magnification and image height of the axial ray at the image plane using Gaussian brackets are given by Equation (1) [23].

(1) $$\begin{array}{l} \left[-z_{0},\frac{1}{f_{1}},-z_{1},\frac{1}{f_{2}},-z_{2},\frac{1}{f_{3}},-z_{3},\frac{1}{f_{4}},-z_{4},\frac{1}{f_{5}}\right]=\frac{1}{m}\\ \left[-z_{0},\frac{1}{f_{1}},-z_{1},\frac{1}{f_{2}},-z_{2},\frac{1}{f_{3}},-z_{3},\frac{1}{f_{4}},-z_{4},\frac{1}{f_{5}},-z_{5}\right]=0 \end{array}$$

In Equation (1), *z*_0_ ~ *z*_5_ and *m* are functions of CAM angle *θ*, and *z*_1_ + *z*_2_ = *T*_12_ and *z*_3_ + *z*_4_ = *T*_34_ are satisfied. *T*_12_ and *T*_34_ are constants that do not change with *θ*. The second equation of Equation (1) is summarized with respect to object distance *z*_0_.

(2) $$z_{0}\left(\theta \right)=\frac{\left[-z_{1}\left(\theta \right),\frac{1}{f_{2}},-T_{12}+z_{1}\left(\theta \right),\frac{1}{f_{3}},-z_{3}\left(\theta \right),\frac{1}{f_{4}},-T_{34}+z_{3}\left(\theta \right),\frac{1}{f_{5}},-z_{5}\right]}{\left[\frac{1}{f_{1}},-z_{1}\left(\theta \right),\frac{1}{f_{2}},-T_{12}+z_{1}\left(\theta \right),\frac{1}{f_{3}},-z_{3}\left(\theta \right),\frac{1}{f_{4}},-T_{34}+z_{3}\left(\theta \right),\frac{1}{f_{5}},-z_{5}\right]}$$

Therefore, if only *z*_3_(*θ*) is determined, *z*_0_(*θ*) can be obtained. By substituting this into Equation (1), *m*(*θ*) can also be obtained. *z*_3_(*θ*) can be determined by spline interpolation from the values given in Table 1. However, angle *θ* satisfying the equation of *z*_0_(*θ*) = 100 can be obtained. Because it is not possible to express Equation (2) analytically for angle *θ*, Equation (2) needs to be solved numerically. The CAM angle for an object distance of 100 mm is ~61.37°. The magnification *m* is about −0.67705 according to the Equation (1).

## 3. Results

### 3.1. Performance Verification of the Multiwavelength Visible-Range-Supported LED Transmission System

**Figure 3:**
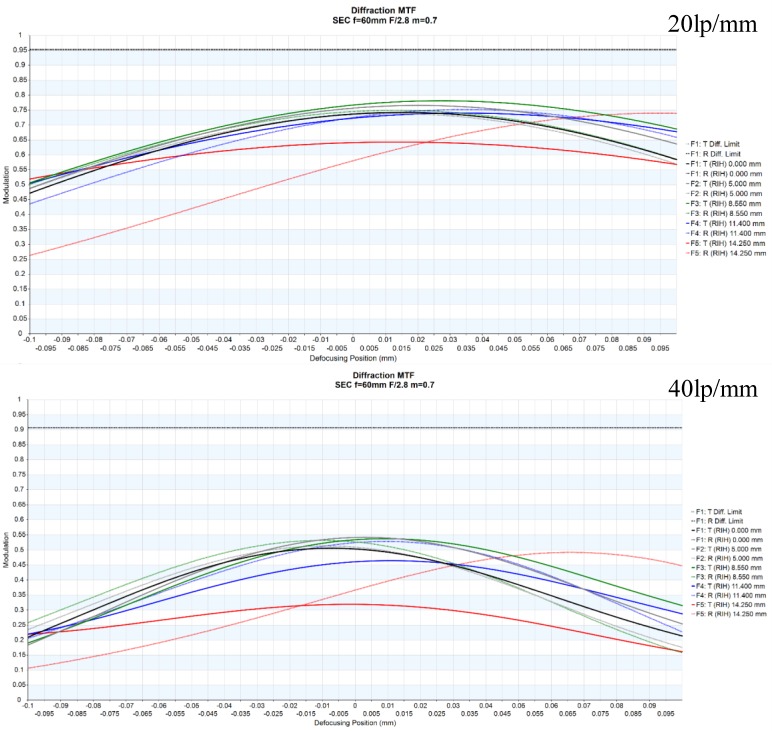
MTF plots for the multiwavelength visible-range-supported LED transmission system.

The modulation transfer function (MTF) is one of the commonly used parameters to show the optical resolutions of an optical system [24]. The MTF graph can demonstrate high-quality optical performance because a high MTF in the optical system cannot be obtained without sufficient compensation for the optical aberration [25]. Therefore, MTF graphs need to be obtained to achieve the proper performance of the custom-designed LED transmission system, as shown in Figure 3. In Figure 3, the upper graph shows the MTF at a spatial frequency of 20 lp/mm, and the lower graph shows the MTF at a spatial frequency of 40 lp/mm. It can be confirmed that there is almost no change in the MTF in the remaining areas, except at the end of the diagonal edge of the sample.

**Figure 4:**
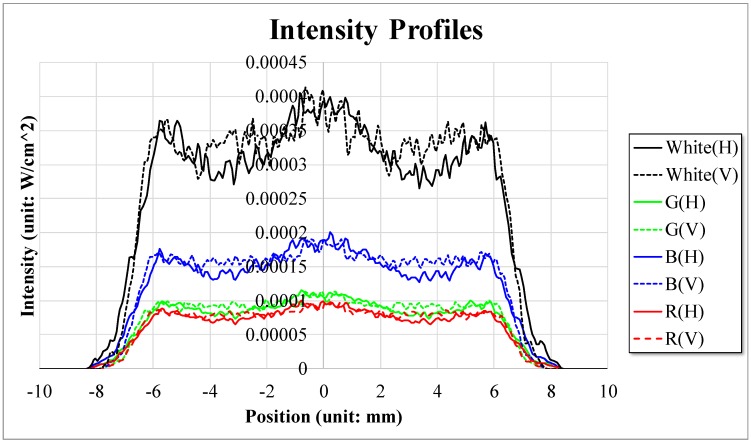
Intensity profile for the multiwavelength visible-range-supported LED transmission system. H and V represent the horizontal and vertical directions.

Figure 4 shows the light intensity profiles of the multiwavelength visible-range-supported LED transmission system. For each plot, the solid line is the intensity profile in the horizontal direction and the dotted line is the intensity profile in the vertical direction.

**Figure 5:**
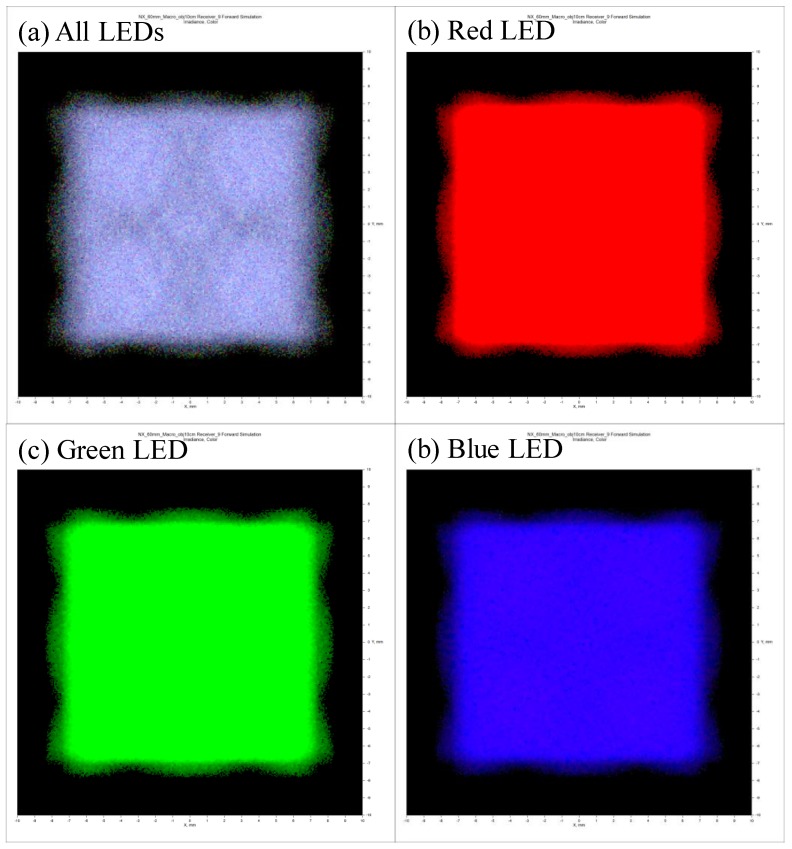
Intensity profiles for the multiwavelength visible-range-supported LED transmission system.

**Figure 6:**
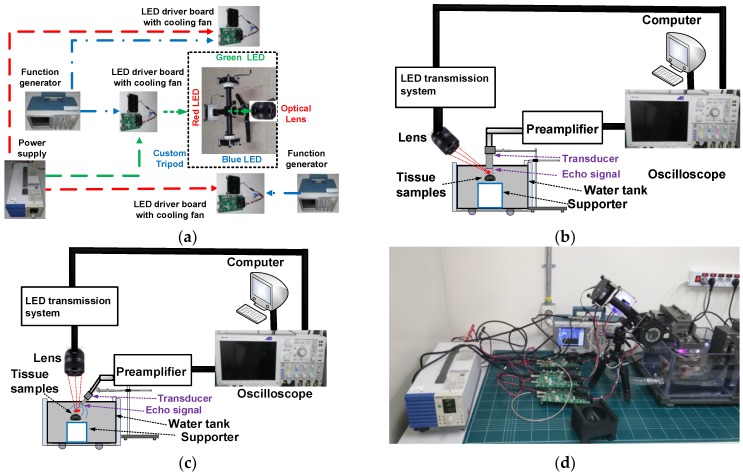
(**a**) Layout of the multiwavelength LED–US instrument with target samples, (**b**) experimental setup when the lens was placed diagonally on the left and the transducer was placed vertically on the right, (**c**) experimental setup when the lens was placed vertically on the left and the transducer was placed diagonally on the right, and (**d**) multiwavelength LED–US experimental instrumentation.

Figure 5 shows the color distribution produced by the designed system. Figure 6a shows the case where all the LEDs are turned on and the sample is illuminated. Figure 6b–d shows the results when only the red LED, green LED or blue LED is turned on. As shown in Figure 6, there is a relatively uniform distribution of light.

### 3.2. Pulse–Echo Responses of Multiwavelength Visible-Range-Supported LED–US Instrument

The A-mode pulse–echo response is one of the typical methods used to evaluate the performances of optical and ultrasound components in ultrasound and opto–ultrasound systems [26]. Figure 6a illustrates the entire structures of the multiwavelength visible-range-supported LED transmission system with an optical lens. Blue (450 nm), green (550 nm) and red (630 nm) LED lights were used as transmission sources. A DC power supply was used to generate 1-kHz and 5-V_p-p_ pulse signals from a function generator (AFG3252C, Tecktronics Inc., Beaverton, OR, USA) trigger three LED driver boards to generate the LED light. In the custom tripod, the LED light converged to generate the light and an optical lens focused the light beam to the desired targets. Figure 6b,c illustrates the experimental setup to verify the feasibility of the developed multiwavelength visible-range-supported LED–US instrument. Eye tissue samples of the *Thunnus obesus* were selected because the samples are large enough (>1 cm) such that we can easily control and focus the LED beam on the samples. In Figure 6b, the LED light through optical lens irradiates the tissue samples of the *Thunnus obesus’* eye vertically, and the ultrasound transducer receives the echo signal at 45°. In Figure 6c, the light through the optical lens irradiates the tissue samples of the *Thunnus obesus’* eye at 45° and the ultrasound transducer receives the echo signal from the samples vertically. An LED system with a specialized design is used as a transmitter. In the transmitter system, a LED (CBT-120, Luminus Devices, Synnyvale, CA, USA) supported by the LED driver (DK-136M-3) and an arbitrary function generator (AFG3252C) were used to feed the LED light to generate the multiwavelength visible-range light. The light illuminated the eye tissue samples of the *Thunnus obesus’* eye, which was mounted on top of the holder in the water tank. After acquiring echo signals from a 10-MHz, 0.5”-diameter, 0.25”-focused ultrasound transducer (V311, Olympus NDT. Inc., Waltham, MA, USA), a preamplifier (AU-1114, Miteq Inc., New York, NY, USA) is then utilized to amplify the weak echo signals, which are then displayed on an oscilloscope (MSO2024B). The echo signals received were processed using envelope detection algorithms using MATLAB (MathWorks Inc., Natick, MA, USA) on a computer to plot the echo signals in the time and frequency domains. The multiwavelength LED–US experimental instrumentation is shown in Figure 6d.

**Figure 7:**
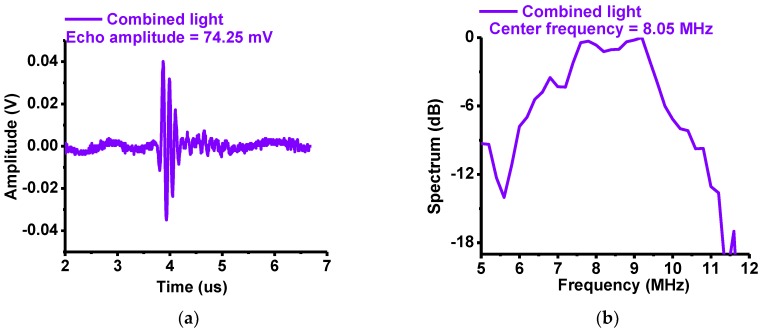
Measured (**a**) peak-to-peak amplitudes and (**b**) center frequencies of the echo signals when the combined red, green and blue LEDs (white light) were irradiated on the eye samples of *Thunnus obesus*.

Figure 7a,b show measured amplitudes and center frequencies, respectively, of the echo signals obtained from the 10-MHz focused ultrasound transducers using eyes of the *Thunnus obesus* as the sample target. The experimental setup is described in Figure 6b. The optical lens was placed diagonally at 45° on the left and the transducer was located vertically in the center. The developed instrument produced white light through combining the red, green and blue LED lights. Each of these colors—red, green, blue and white (combined) LED lights—were shone on the *Thunnus obesus’* eye samples through the optical lens, and echo signals generated from the samples were detected by the ultrasound transducers. When using red, green, blue and white LED lights, the measured echo amplitudes were 28.32, 24.54, 27.51 and 74.54 mV, respectively, and the center frequencies were 8.31, 8.25, 8.54 and 8.05 MHz, respectively.

**Figure 8:**
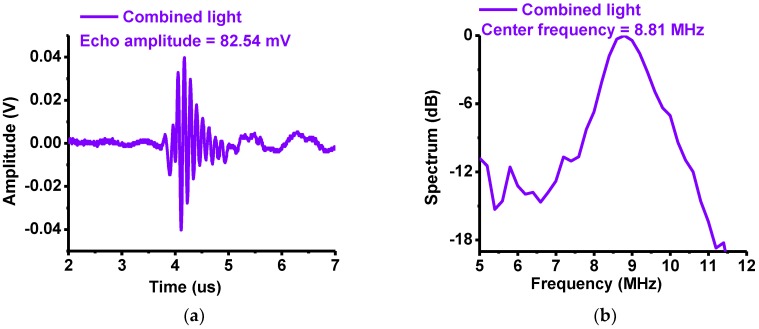
Measured (**a**) peak-to-peak amplitudes and (**b**) center frequencies of the echo signals when the combined red, green and blue LEDs (white light) were irradiated on the eye sample of *Thunnus obesus*.

Figure 8a,b show the measured amplitudes and center frequencies, respectively, of the echo signals obtained from the 10-MHz focused ultrasound transducers when the samples are eyes of the *Thunnus obesus*. The experimental setup is described in Figure 6c. The lens was placed vertically in the center and the transducer was diagonally at 45° in the right. When the red, green and blue LEDs and the combined LEDs were used, the measured echo amplitudes were 33.53, 34.92, 38.74 and 82.54 mV, respectively, and the center frequencies were 9.02, 9.05, 9.21 and 8.81 MHz, respectively. The amplitudes of the echo signals were slightly higher when the lens was placed vertically compared to the case when the lens was placed diagonally because the light intensity when the lens was placed vertically was a little stronger.

## 4. Conclusions

A new multiwavelength-supported LED–US instrument with a custom-designed optical setup was proposed to generate multiwavelength optical light (400–600 nm) with low optical aberration, and to generate acoustic signals. To assess the instrument’s capability, an integrated red, green and blue LED source was used to produce white LED light. The LED source generates sufficient light intensity and distribution from a 10-cm distance. In the instrument, the LED transmission system provides a reasonably flat modulation transfer function at spatial frequencies of 20 and 40 lp/mm except at the end of the diagonal edge of the sample. Pulse–echo responses using *Thunnus obesus* eye samples were obtained to verify the capability of the designed LED–US instrument with different configurations. When the optical lens was placed diagonally at 45° on the left and the transducer was placed vertically in the center, the echo amplitudes received from the ultrasound transducer were 28.32, 24.54, 27.51 and 74.54 mV, respectively, and the center frequencies were 8.31, 8.25, 8.54 and 8.05 MHz, respectively, when producing red, green, blue and white LED light. Additionally, when the optical system was located vertically in the center and the transducer was placed on the right, the echo amplitudes received from ultrasound transducer were 33.53, 34.92, 38.74 and 82.54 mV, respectively, and the center frequencies were 9.02, 9.05, 9.21 and 8.81 MHz, respectively, when producing red, green, blue and white LED light. Therefore, this instrument is able to combine LEDs of different spectra to cover a wide range of wavelengths in the visible-light spectrum. We believe that this is the first step towards devising a complete multiwavelength adjustable-focus LED–US instrument for monitoring human eyes.

The developed multiwavelength range-supported LED–US instrument can possibly be utilized to assess properties of the eye via chromophores such as hemoglobin and melanin. Additionally, adjustable focal distances with multiwavelength ranges will be useful for patients with different eye pupil sizes.

## Figures and Tables

**Table 1:** Loci of optical system for multiwavelength visible-range-supported LED transmission system. The units of the distance and CAM angle are mm and degrees, respectively.

CAM Angle (*θ*)	Magnification (*m*)	Object to 1st Surface (*z*_0_)	5th Surface to 6th Surface (*z*_1_)	10th Surface to 11th Surface (*z*_2_)	15th Surface to 17th Surface (*z*_3_)	21st Surface to 22nd Surface (*z*_4_)	23rd Surface to Image (*z*_5_)
0	0.000000	infinity	2.000000	11.289500	20.485869	10.922631	23.015842
10	−0.1602570	362.66261	3.042602	10.246898	16.839850	14.568650	23.015842
20	−0.2738123	215.09018	4.085205	9.204295	14.639113	16.769387	23.015842
30	−0.3793376	158.81184	5.127810	8.161690	12.638163	18.770337	23.015842
40	−0.4784159	129.86053	6.170413	7.119087	10.844003	20.564497	23.015842
50	−0.5724333	112.66637	7.213015	6.076485	9.260316	22.148184	23.015842
60	−0.6643599	101.28187	8.255619	5.033881	7.825628	23.582872	23.015842
70	−0.7586855	92.932035	9.298222	3.991278	6.420389	24.988111	23.015842
80	−0.8605840	86.269697	10.340826	2.948674	4.908803	26.499697	23.015842
90	0.973438	80.799997	11.38343	1.906070	3.237413	28.171087	23.015842
